# Biodiversity and Archeological Conservation Connected: Aragonite Shell Middens Increase Plant Diversity

**DOI:** 10.1093/biosci/bit038

**Published:** 2014-02-04

**Authors:** Sula E. Vanderplank, Sergio Mata, Exequiel Ezcurra

**Affiliations:** Sula E. Vanderplank is affiliated with the Botanical Research Institute of Texas, in Fort Worth. Sergio Mata is affiliated with the Society of Native Plants of Baja California, in Ensenada, Baja California, Mexico. Exequiel Ezcurra is affiliated with the Department of Plant Sciences at the University of California, Riverside sula.vanderplank@gmail.com.

**Keywords:** *Tivela*, cultural landscape, Baja California, Mexico, coastal

## Abstract

Natural and cultural heritage sites frequently have nonoverlapping or even conflicting conservation priorities, because human impacts have often resulted in local extirpations and reduced levels of native biodiversity. Over thousands of years, the predictable winter rains of northwestern Baja California have weathered calcium from the clam shells deposited by indigenous peoples in middens along the coast. The release of this calcium has changed soil properties, remediated sodic and saline soils, and resulted in a unique microhabitat that harbors plant assemblages very different from those of the surrounding matrix. Native plant biodiversity and landscape heterogeneity are significantly increased on the anthropogenic soils of these shell middens. Protection of this cultural landscape in the Anthropocene will further both archeological and biodiversity conservation in these anthropogenic footprints from the Holocene. Along these coasts, natural and cultural heritage priorities are overlapping and mutually beneficial.

**The preservation of archeological sites does not** always overlap with the conservation of biodiversity. At the most basic level, the United Nations Educational, Scientific, and Cultural Organization separates cultural heritage sites and natural heritage sites: Of 981 heritage sites, 759 are cultural, 193 natural, and only 29 (2.65%) have mixed properties (*whc.unesco.org/en/list*). Cultural conservation and biodiversity conservation have overlapped in the sustainable use of natural resources (Timmer and Juma 2005), but shared targets of elevated conservation importance for both archeological and biodiversity priorities are still few.

People have harvested coastal resources for more than 150,000 years and have affected populations of shellfish dating back more than 23,000 years (Jackson JBC et al. 2001, Rick and Erlandson 2009). Along the coasts of the Americas, hunter–gatherers exploited coastal ecosystems, leaving behind shell mounds, or *middens*, which form microhabitats for distinct biotic communities. Humans were significant components of coastal ecosystems for millennia, blurring the boundaries between the natural and anthropogenic worlds deep into human prehistory (Rick and Erlandson 2009).

The Pacific coast of North America is littered with shell middens from a variety of indigenous peoples, whose long-term occupation of the coast resulted in heavy marine deposits on land. The Seri people (one of the most robust and intact indigenous communities in northern Mexico and one that retains considerable traditional knowledge) apply common indigenous names to over 150 species of mollusks and still rely heavily on shellfish as part of their subsistence (Bertsch and Moser Marlett 2011). Coastal disturbance and development has destroyed many coastal middens in California, but many still remain in Baja California, Mexico, although development pressure is increasing (Moore 2006).

Human inhabitation of the Baja California Peninsula, often referred to as *the forgotten peninsula* by archeologists, began more than 11,000 years ago (Laylander and Moore 2006, Des Lauriers 2010). Occupation was transient and migratory, involving large distances and small (fewer than 30 people) family groups (Hyland 1997, Moore 2012). People moved freely from mountains to sea, often relying heavily on marine resources for protein. In Baja California, the harvesting of shellfish (clams, mussels, and abalone) is evidenced in the presence of large middens, defined by the presence of soil, discarded shells, archeological artifacts, and sometimes charcoal (Mudie and Lelievre 2013). After thousands of years, many of these shell middens have become mounds of calcium-rich soil that provide new habitats for plants (figure 1; Felger 2007).

**Figure 1.**
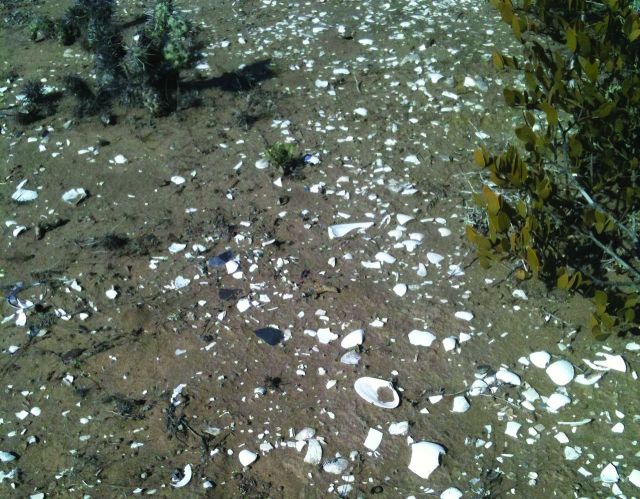
Aragonite shell middens in San Quintín allow the growth of nonhalophytic plants, such as jojoba and cholla, which do not prosper in the saline soils of the surrounding matrix. Note the decomposed shells of pismo clams and the flakes from hand tools visible on the soil surface. Photograph: Sula E. Vanderplank.

The Paipai and Kiliwa people of northwestern Baja California (among the last speakers of indigenous languages in the region) did not leave pyramids, temples, or permanent structures (Meigs 1935). Their homes were often ephemeral and their lifestyle transitory (Moore 1999, 2012, Figueroa Beltrán 2010), but their middens, dotted across the landscape and often many kilometers inland (Meigs 1935, 1938, Moore 2001), are some of the most significant archeological sites in the region. Moore (1999, 2001) dated many of the middens in the San Quintín region and recorded regular occupation from the first half of the sixth millennium into historic times, with the majority of the middens being 3000–5000 years old.

The shell middens found in northwestern Baja California can broadly be separated into two types on the basis of shell type. Calcium carbonate crystals in shells are embedded within an organic matrix and occur in two forms: calcite and aragonite. Nacre-forming calcitic species are found chiefly on exposed rocky foreshores (e.g., the California mussel [*Mytilus californianus*] and the black abalone [*Haliotis cracherodii*]). Aragonitic species are found mostly on sandy beaches with strong surf (predominantly, the Pismo clam [*Tivela stultorum*]); their shells have lamellar layers of aragonite but neither calcite nor nacre (Coan et al. 2000). Calcitic shells (which sometimes include aragonitic layers) are more stable, and the shells degrade relatively slowly, whereas aragonitic species have shells that decompose more readily and release calcium carbonate into the environment (Harper 2000).

In the Mediterranean-climate region of Baja California, where winter rainfall is sufficient for soil development and the weathering of shells, the effect of these prehistoric middens on contemporary vegetation is noticeable along low, sedimentary coasts (Vanderplank 2010). Meigs (1938) suggested that studies on the vegetation of the middens of northwestern Baja California could uncover patterns of movements of people historically or successional patterns in the flora, because he observed the presence of *Ambrosia chenopodiifolia* (syn. *Franseria chenopodiifolia*) on recent midden sites. His observation may be related to the remains of agave roasting pits, because this species often dominates on burnt soil. Similar vegetation changes between middens and the surrounding vegetation matrix are not so clearly observed along rocky shores, where middens are made up mostly of calcitic shells.

The leading hypothesis of the present study is that the concentrated prehistoric deposition of aragonitic shells affects the contemporary distribution of native plants. To investigate the relationship between current plant distributions and past human occupation, we compared the flora of two major midden complexes in northwestern Baja California with that of the surrounding soil matrices. We further hypothesize that historical human activity has increased environmental heterogeneity, which has resulted in increased plant diversity and a more complex species assemblage. We also analyze the conservation value of past human disturbances by investigating whether human activity has enhanced landscape heterogeneity and native plant species richness, which would make conservation priorities for biodiversity and archeological sites overlapping and mutually beneficial.

## Site selection

**Figure 2.**
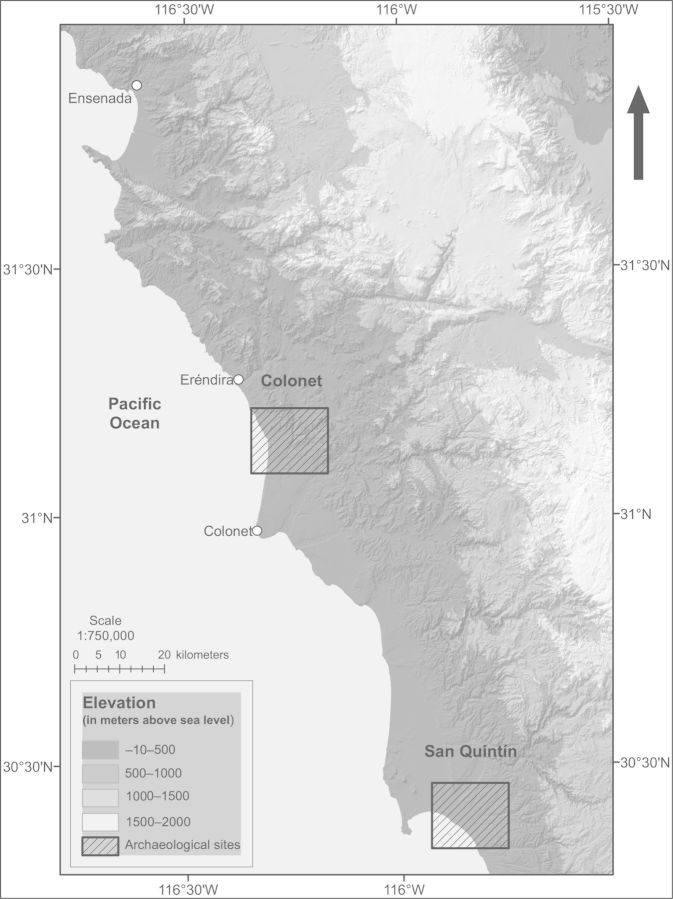
Study regions (Colonet and San Quintín) on the Pacific coast of Baja California.

Two extensive midden complexes from the historical territories of the Paipai and Kiliwa people in northwestern Baja California were identified at sites where extensive archeological investigation had already been conducted (figure 2; Colonet and San Quintín). The exact locations of the study sites are not presented here, at the request of the National Institute of Anthropology and History of Mexico, because of the sensitive nature of the archeological sites. Three midden complexes were selected in the Colonet region, for a total of 11 middens in that region, and four were selected in the San Quintín region, for a total of 15 middens in that region. The mean annual precipitation and mean temperature, respectively, are 250 millimeters (mm) and 17 degrees Celsius (°C) in Colonet and 140 mm and 16°C in San Quintín (Ruiz Corral et al. 2006). Midden sampling was conducted with specific prerequisites for site selection. The criteria included a suitable distance from the ocean (i.e., not directly adjacent), with matrix vegetation on all sides; sizable ancient middens; aragonite shell middens; stable plant communities present; and the presence of a fine-grain soil matrix.

## Floristic analysis

Two-meter (m)–wide belt transects were run parallel to the coast (north to south) along the entire length of each midden (20–200 m in length). Matrix control transects of the same length were laid both coastward and inland of each midden to control for edge effects and distance from the coast. For each belt transect, all perennial species were listed (and their abundance was quantified using cover estimates). The percentage of aragonite shell visible at the midden surface was recorded, together with the percentage of bare ground, as a measure of total vegetative cover.

To examine the distribution of perennial species across all samples and both sites, a nonstandardized principal components analysis (PCA) was conducted for the two sites individually and combined, using each transect as a sampling unit. D. A. Jackson's (1993) broken-stick distribution model was used to test for significant axes. The overall species ­richness on and off the middens for each sample unit was calculated and averaged across transects. The preference of any given species for the midden habitat over the surrounding matrix was evaluated by calculating the species’ frequency in the middens and outside the middens and comparing both binomial frequencies by means of a *t*-test using a Bonferroni correction for multiple comparisons (Sokal and Rohlf 2011).

## Soil analysis

A saturated paste extract was obtained from soil samples on and off the middens at three sites in Colonet and four sites in San Quintín. In the extract solution, electrical conductivity (EC) was measured with a portable EC meter, and water-soluble cations were quantified using inductively coupled plasma (ICP) analysis in the University of California, Riverside, soil science laboratory.

To verify the accuracy of EC as an estimation of the total dissolved salts in the soil solution, we correlated our EC measures with the ICP estimation of total cations and found an almost perfect linear relationship between the two measures (*r* = .996, *p* < .0001, *n* = 14). To test for soil sodicity, we transformed our cation data set to sodium adsorption ratio (SAR) values, using the standard soil-science formula

\begin{equation*}
{\rm SAR} = Na/((Ca + Mg)/2)^{1/2} ,
\end{equation*}

where the variables *Na*, *Ca*, and *Mg* represent the concentrations of sodium, calcium, and magnesium, respectively, in milliequivalents per liter. The SAR index measures the activity of sodium as a dispersant on soil clay particles relative to the flocculation effect of the bivalent ions calcium and magnesium.

## Floristic data

Despite the proximity of the two study regions, they are almost entirely different in their floristic composition (both on the middens and in the surrounding matrix). The only species found significantly on both midden complexes was *Marah macrocarpa*, an abundant globose succulent with a wide geographic range. The almost complete floristic disjunction between the two regions was captured by the PCA of the whole data matrix, in which the first axis (explaining 37% of the total variance) separated the two study regions into two very distinct clusters, which highlights the fact that the plants of the middens have no commonalities between the two regions.

**Figure 3.**
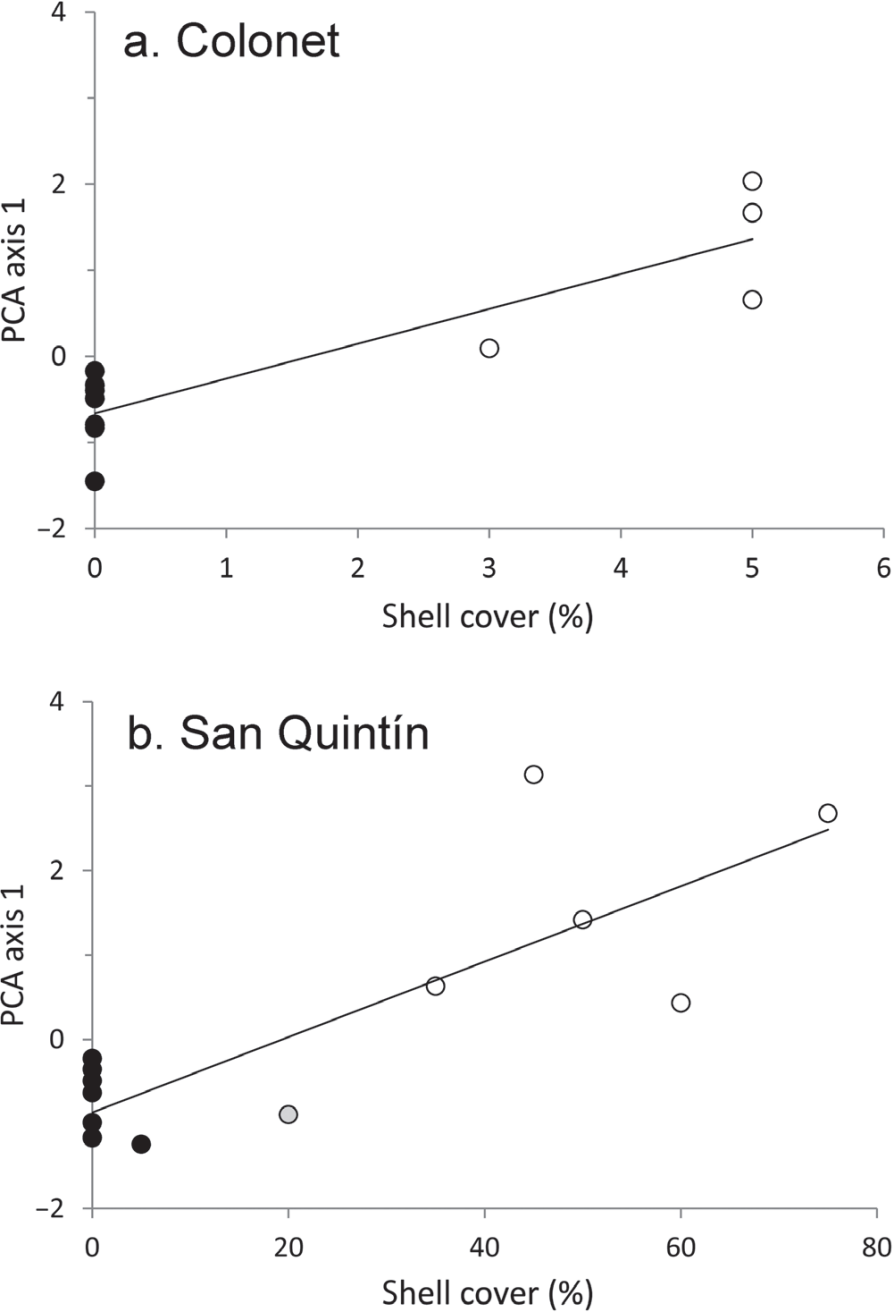
Correlation between floristic variation (the first principal components analysis [PCA] axis) and the percentage of surface shell cover for (a) Colonet (*r* = .89, *p* < .0001) and (b) San Quintín (*r* = .84, *p* < .0001). The matrix (off-midden) transects are indicated by black dots and the on-midden transects by white dots. An atypical midden transect with low shell cover is indicated by a gray dot. The species significantly associated with the floristic axes (i.e., the surrounding matrix and midden species for each region) are listed in table 1.

The separate PCA for each region had only one statistically significant axis, which explained 30% of total floristic variance in Colonet and 34% in San Quintín. In both regions, these floristic axes correlated very strongly with the amount of shell cover in the soil (*r* = .89, *p* = .0003 in Colonet; *r* = .84, *p* = .0001 in San Quintín; figure 3, table 1). Floristic variation, summarized by the PCA axes, was strongly correlated with the amount of shell at the site.

**Table 1.** Species with significant affinities for middens or surrounding matrix sites in Colonet and San Quintín.

Surrounding matrix species		Midden species	
San Quintín	Colonet	San Quintín	Colonet
*Arthrocnemum subterminale*	*Acmispon glaber*	*Aesculus parryi*	*Astragalus trichopodus*
*Atriplex barclayana*	*Dudleya ingens*	*Artemisia californica*	*Encelia californica*
*Frankenia salina*	*Eriogonum fasciculatum*	*Astragalus trichopodus*	*Euphorbia misera*
*Juncus acutus*	*Hazardia ferrisiae*	*Atriplex canescens*	*Marah macrocarpa*
*Salicornia pacifica*	*Rosa minutifolia*	*Atriplex julacea*	*Rhus integrifolia*
		*Cylindropuntia californica*	
		*Cylindropuntia prolifera*	
		*Dudleya cultrata*	
		*Echinocereus maritimus*	
		*Ephedra californica*	
		*Ferocactus fordii*	
		*Gambelia juncea*	
		*Lycium californicum*	
		*Mammillaria dioica*	
		*Mirabilis laevis*	
		*Sphaeralcea ambigua*	

*Note:* We selected species that had significantly high or low axis loadings in the principal components analysis and that differed significantly in their frequency in middens as compared with the matrix according to a *t*-test on the binomial frequencies. In order to avoid errors derived from multiple comparisons, we used a Bonferroni correction with *α* = .001 for San Quintín and *α* = .003 for Colonet.

Ordering the species by their PCA scores, we obtained for both regions a list of the species that preferentially colonize middens on one hand and a list of those that are found chiefly off middens on the other (see supplemental table S1 for Colonet and table S2 for San Quintín). According to the *t*-tests on their binomial frequencies on and off middens, 16 species were found in San Quintín showing a significant preference for middens, whereas 5 showed a significant preference for the surrounding matrix. In Colonet, in contrast, only five species were found with a significant preference for middens and five with a preference for the surrounding matrix.

**Figure 4:**
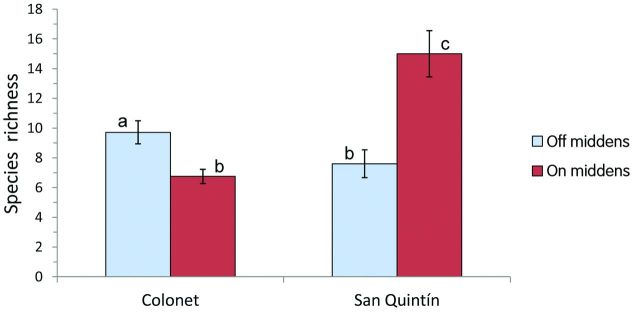
Floristic richness on and off middens in Colonet and San Quintín. The bars labeled by different letters are significantly different according to a *t*-test (α = .01). The error bars represent the standard error.

Significant differences in species richness on and off middens were found at both sites. San Quintín had the highest level of species richness on the middens themselves, whereas the Colonet middens had lower levels of richness than the surrounding matrix (figure 4). Both sites host a flora on the middens very different from that in their surrounding ­vegetation matrix, and this significantly demonstrates the effect of middens on overall species diversity in both regions.

## Site-specific differences

**Figure 5.**
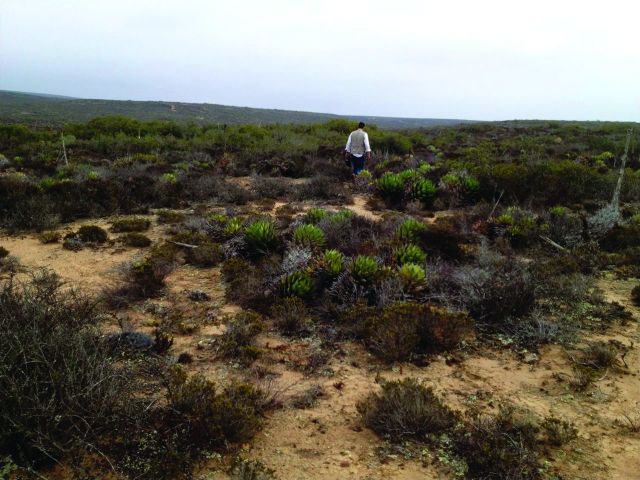
Distinct vegetation differences are visible when looking from the surrounding matrix toward the taller vegetation found on a Colonet midden. Photograph: Sula E. Vanderplank.

Each region had midden complexes with distinct characteristics. The San Quintín middens were raised up to 3 m higher than the surrounding matrix, apparently from sand trapped by shell piles. The shells in San Quintín are almost exclusively aragonite, and the vegetation is taller than that on the surrounding matrix (see figure 5). The Colonet middens are almost flat, with much lower quantities of shell visible at the soil surface and a small percentage of calcite shells visible in addition to aragonite shells. The soils of the surrounding matrix appear to have more clay than the silty soils of San Quintín, and burnt soils suggest that agave roasting may have taken place at some midden sites, although it may also be the result of recent fire. The vegetative cover on the middens at Colonet is also distinctly taller than that in the surrounding matrix. Despite lower levels of species richness on the Colonet middens than on the surrounding matrix, the vegetative cover was often seen to be highest on the midden (data available on request).

## Soil data

**Figure 6.**
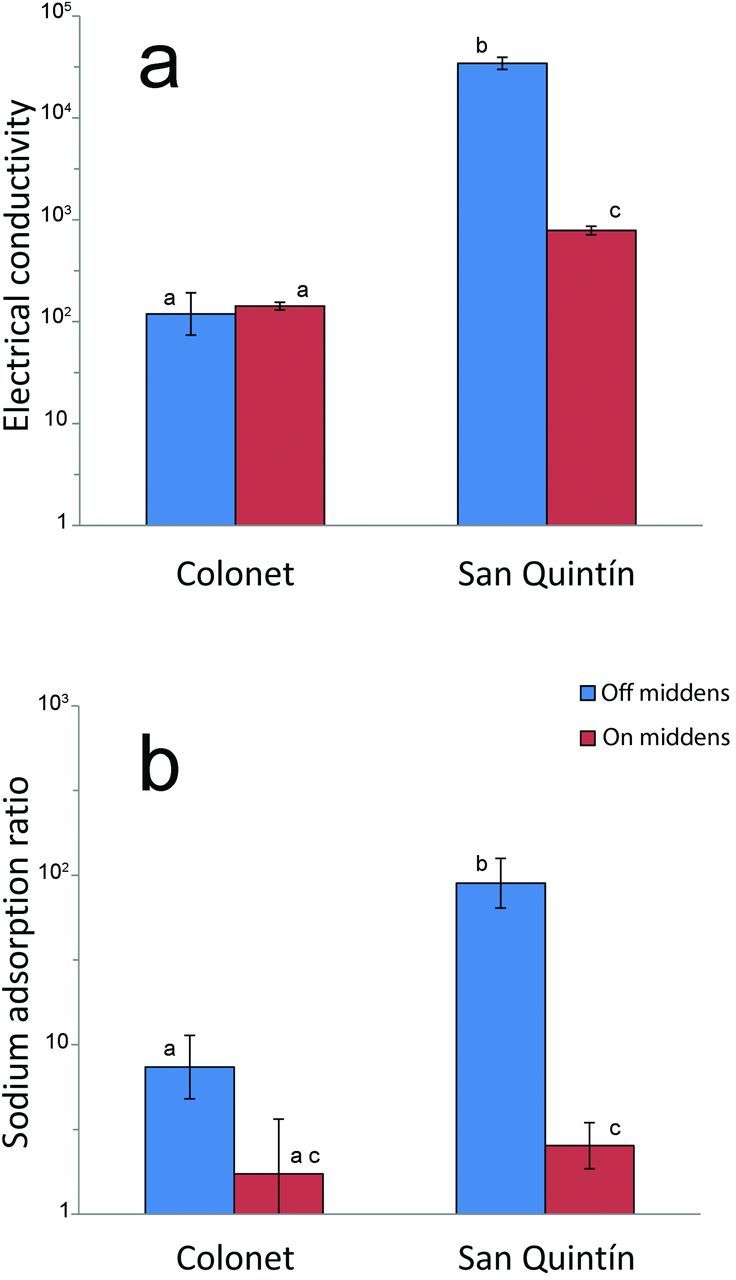
(a) Electrical conductivity (in microsiemens per centimeter) and (b) sodium adsorption ratio off and on middens in Colonet and San Quintín. In order to keep all data points within a visually comparable scale, the vertical axis was log-transformed in both plots. In each graph, the bars labeled by different letters are significantly different according to a *t*-test (*α* = .01). The error bars represent the standard error.

In the low-lying, floodable soils of San Quintín, the presence of shell middens had a very large effect on soil salinity. The surrounding matrix soils were strongly saline, with a mean conductivity of 34,400 microsiemens per centimeter (mS/cm), whereas the midden soils were nonsaline, with a mean conductivity of around 790 mS/cm (in agricultural science, the conventionally accepted threshold for saline soils is 4000 mS/cm). In the higher-elevation mesa of Colonet, removed from the flood zone, no significant differences were observed in EC between the surrounding matrix and the midden soils, both of which showed an average of around 120–140 mS/cm (figure 6).

Sodicity in the soil, measured as the SAR of the saturated paste extract solution, showed a similar trend. In the tidally floodable matrix soils of San Quintín, sodicity was extremely high, with a mean SAR value of 89, whereas the midden soils were nonsodic, with mean SAR of around 2.5 (the conventionally accepted SAR threshold for sodic soils is 13). In Colonet, a generally similar but less pronounced pattern was observed. The mean SAR value was 7.4 in the surrounding matrix soils and 1.7 in the middens, but this difference was only marginally significant (*p* = .1), because of relatively high standard errors.

## Calcium in coastal soils

The beneficial effects of calcium added onto saline and sodic soils are well known (Buol et al. 2011). The bivalent calcium cations displace other soil cations and significantly change soil characteristics, flocculating soil particles, giving the soil a porous structure, and promoting the leaching of salts. In contrast, sodium cations cause soil particles to deflocculate and disperse, giving sodic soils a lack of internal structure and poor drainage that create difficult conditions for plant growth and that promote salinization (Brady and Weil 2007). The effect of aragonite shells as a soil amendment agent was strikingly visible in the tidally flooded soils, where both dissolved salts and sodicity in the middens were only a small fraction (2%–3%) of those of the concentration in the surrounding matrix.

Our data suggest that the release of calcium as a causative agent of soil modification is the main factor driving the differential colonization of middens by native plants. The differences observed between our two study regions show that the impacts of the middens, themselves, are matrix dependent, and the effect of calcium varies with the original soil conditions. The adjacent coastal desert regions in the peninsula also harbor extensive middens, but the lack of soil development (sandy soils) and steady rainfall for weathering have failed to produce similarly detectable effects during the same time.

## Floristic patterns

The surrounding matrix species in San Quintín are all salt-marsh species. The surrounding matrix species in Colonet are maritime succulent scrub species, with a high number of local endemics. The midden species in San Quintín are maritime succulent scrub, with a strong desert influence and a surprising number of succulent plants, perhaps as a result of the sandy soil of the midden. The midden species in Colonet are Mediterranean-climate species that are abundant elsewhere in the region. The middens in the Colonet region were sometimes adjacent to vernal pools, which are another key conservation target for biological diversity.

Statistically, the effect of the shell middens on vegetation is clearly nested within the sites: Although the presence of middens has induced significant floristic changes in both sites, these changes are site specific. For example, there are opposite trends in species richness between the two sites. In San Quintín, richness is greatly elevated on the middens, whereas in Colonet, richness is lower on the middens. The addition of calcium had a divergent effect on the soil matrix in these two communities and on the present vegetation. It is unequivocal that the human harvest of marine invertebrates leaves a trail of environmental transformation that increases native diversity and landscape heterogeneity. At the same time, species colonization of middens in different habitats is not predictable, because each soil type responds differently to the addition of calcium, which results in different plant communities. The deposition of aragonite shells did not promote the occurrence of calciphiles (calcium-loving species). Instead, it is the formation of a more heterogeneous landscape, driven by the footprint of prehistoric human activities, that promotes species richness at each site.

## Soil–vegetation interactions

Because of the nested nature of the effect of middens within sites, considerable differences were found in the middens of the two study regions, including microterrain (raised or flat), the quantity of shells, soil type, and salinity. Although, as a general rule, we can conclude that the presence of shell middens increases environmental heterogeneity and, therefore, plant diversity, the process seems to be mechanistically different at the two sites. The shared effect of shells at the two sites allows us to discount the mounding effects as the single source of the observed floristic change. The shell quantities at the two regions varied markedly, and the impact was different: Small amounts of surface shell (3%–5%) were enough to significantly affect floristic composition at Colonet, whereas more than 20% shell at San Quintín was necessary to yield a marked floristic difference from the surrounding matrix. The role of factors such as the type and duration of human occupation, whether cooking fires took place and roasting pits were made, and the age of the individual middens is unclear. The lack of dated middens at Colonet prohibits an age comparison, but Figueroa Beltrán (2010) hypothesized that site occupation may have been more impermanent and transient than at adjacent regions because of the lack of freshwater.

The arrival of the Anthropocene, a new geological epoch marked by human domination of Earth systems, is reflected in the shell middens studied here. Deposited during the Holocene, the shells have been weathered, and new anthropogenic soils have formed that show unique soil signatures and plant communities. These soils, which resulted from human activity, have diversified the cultural landscape and increased vegetation heterogeneity. Cultural sites in this context therefore increase native plant biodiversity, and the Anthropocene can be expected to show surprising impacts on our native flora, given sufficient time.

## The prehistoric footprint

The impact of aragonite shell middens on soil conditions and plant communities is evidence that the historical activity of indigenous people has augmented the native plant biodiversity. The cultural landscape is more heterogeneous, favoring unique plant assemblages within the surrounding matrix–midden mosaic. Recent studies have shown that, in the San Quintín region, the percentage of endemic and rare plants is strongly correlated with overall plant species richness (Vanderplank 2010). Therefore, we can expect an increase in the number of endemic and rare plants that grow in the cultural landscape of the broader region because of increases in species diversity provided by the midden mosaic.

Previous evidence of human impacts increasing biodiversity has been reported only in light of indigenous management of the land (e.g., burning or gardening; Gadgil et al. 1993). This is the first example of past human activities inadvertently benefiting terrestrial biodiversity as a result of harvested marine life and altered soils. In the future, the vegetation of the middens may continue to change with increasing amounts of calcium carbonate being released into the soil as shells decompose. The middens will continue to maintain a flora distinct from that of the surrounding matrix and will maintain landscape heterogeneity and native plant richness, even if the floristic composition varies through time. Long-term monitoring of middens may inform our understanding of plant ecology in soils in which shells are being continually eroded. Over longer timescales, we may see a similar effect in middens of calcite shells, which decompose more slowly than aragonite shells.

Shell middens in northwestern Mexico have been previously proposed to have great potential for ecotourism and educational purposes (Téllez-Duarte et al. 2005). The value of conserving biodiversity in areas of traditional management and simultaneously conserving native cultures has long been recognized (Furze et al. 1996, Timmer and Juma 2005), but the conservation of abandoned archeological sites has been more challenging. The surviving indigenous people of Baja California have been largely displaced (relocated to the north). The Kiliwa people, who inhabited San Quintín, now number approximately 50 individuals, living along Arroyo León, south of Valle de la Trinidad, with just 5 native speakers. The Paipai, who inhabited Colonet, now number approximately 350 people, living in two communities (in Valle de la Trinidad and Sierra de Juárez; *http://bajacomunidad.org/tribes*). To preserve their history and the rich biodiversity that results from their ancestral stewardship, we hereby propose that additional protection and vigilance be given to the shell middens of northwestern Baja California.

**Figure 7.**
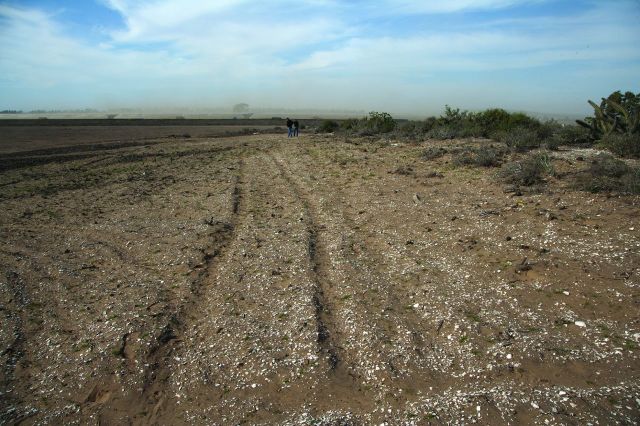
Following the precedent of neighboring California, the coastal middens of the Pacific coast of Baja California are being destroyed by expanding agriculture and urbanization. Photograph: Sula E. Vanderplank.

In conclusion, our study shows that, in these regions, priorities for the conservation of archeological sites and terrestrial biodiversity overlap and are complementary in their targets (figure 7). Anthropogenic soils can increase native plant biodiversity and landscape heterogeneity. Conservation efforts focused on the local natural and cultural heritages will mutually benefit from the increased protection of archeological shell middens.

## Supplementary Material

SUPPORTING INFORMATION

## Supplemental material

The supplemental material is available online at http://bioscience.oxfordjournals.org/lookup/suppl/doi:10.1093/biosci/bit038/-/DC1.
