# Supplementary material for: Biodiversity and Archeological Conservation Connected: Aragonite Shell Middens Increase Plant Diversity
Source: Bioscience. 2014 Feb 4;64(3):202–9. doi: 10.1093/biosci/bit038 (PMC4776670; doi:10.1093/biosci/bit038)
Supplement: SUPPORTING INFORMATION [file supp_64_3_202__index.html]

Biodiversity and Archeological Conservation Connected: Aragonite Shell Middens Increase Plant Diversity — SUPPORTING INFORMATION 

# Biodiversity and Archeological Conservation Connected: Aragonite Shell Middens Increase Plant Diversity

## HTML Page - index.htslp

**Files in this Data Supplement:**

- Data Supplement
