# Supplementary material for: Biodiversity and Archeological Conservation Connected: Aragonite Shell Middens Increase Plant Diversity
Source: Bioscience. 2014 Feb 4;64(3):202–9. doi: 10.1093/biosci/bit038 (PMC4776670; doi:10.1093/biosci/bit038)
Supplement: SUPPORTING INFORMATION [file supp_bit038_FINAL-Vanderplank_Supplement.doc]

Table S1. Species frequencies in and out of middens at Colonet. Preference is shown in or out of middens only for taxa that were statistically significant according to a *t*-test.

| Family | Scientific name | frec out | frec in | Preference |
| --- | --- | --- | --- | --- |
| Agavaceae | *Agave shawii* | 0.9 | 0.8 |  |
| Anacardiaceae | *Rhus integrifolia* | 0.3 | 0.8 | **in** |
| Asteraceae | *Hazardia ferrisiae* | 0.4 | 0.0 | **out** |
| Asteraceae | *Artemisia california* | 0.9 | 0.8 |  |
| Asteraceae | *Isocoma menziesii* | 0.1 | 0.3 |  |
| Asteraceae | *Encelia californica* | 0.0 | 0.8 | **in** |
| Cactaceae | *Echinocereus maritimus* | 0.3 | 0.0 |  |
| Cactaceae | *Ferocactus viridescens* | 0.6 | 0.0 | **out** |
| Cactaceae | *Mammillaria dioica* | 0.6 | 0.3 |  |
| Crassulaceae | *Dudleya ingens* | 1.0 | 0.5 | **out** |
| Crassulaceae | *Dudleya attenuata* | 0.3 | 0.0 |  |
| Cucurbitaceae | *Marah macrocarpa* | 0.0 | 0.3 | **in** |
| Ephedraceae | *Ephedra californica* | 0.4 | 0.0 | **out** |
| Euphorbiaceae | *Euphorbia misera* | 0.0 | 0.5 | **in** |
| Fabaceae | *Acmispon glaber* | 1.0 | 0.3 | **out** |
| Fabaceae | *Astragalus trichopodus* | 0.0 | 0.3 | **in** |
| Polygonaceae | *Eriogonum fasciculatum* | 1.0 | 0.3 | **out** |
| Polygonaceae | *Eriogonum fastigiatum* | 0.1 | 0.0 |  |
| Rosaceae | *Rosa minutifolia* | 1.0 | 0.5 | **out** |
| Simmondsiaceae | *Simmondsia chinensis* | 0.9 | 0.8 |  |

Table S2. Species frequencies in and out of middens at San Quintín. Preference is shown in or out of middens only for taxa that were statistically significant according to a *t*-test.

| Family | Taxon | frec out | frec in | Preference |
| --- | --- | --- | --- | --- |
| Anacardiaceae | *Toxicodendron diversilobum* | 0.1 | 0 |  |
| Anacardiaceae | *Rhus integrifolia* | 0 | 0.2 | **in** |
| Asteraceae | *Isocoma menziesii* | 0.7 | 0.8 |  |
| Asteraceae | *Helianthus niveus* | 0 | 0.2 | **in** |
| Asteraceae | *Ambrosia chenopodiifolia* | 0 | 0.2 | **in** |
| Asteraceae | *Artemisia california* | 0 | 0.4 | **in** |
| Cactaceae | *Lophocereus schottii* | 0 | 0.2 | **in** |
| Cactaceae | *Stenocereus gummosus* | 0 | 0.2 | **in** |
| Cactaceae | *Echinocereus maritimus* | 0 | 0.4 | **in** |
| Cactaceae | *Cylindropuntia prolifera* | 0 | 0.4 | **in** |
| Cactaceae | *Ferocactus fordii* | 0 | 0.6 | **in** |
| Cactaceae | *Cylindropuntia californica* | 0 | 0.6 | **in** |
| Cactaceae | *Mammillaria dioica* | 0 | 0.4 | **in** |
| Chenopodiaceae | *Salicornia pacifica* | 0.9 | 0.4 | **out** |
| Chenopodiaceae | *Atriplex watsonii* | 0.5 | 0.4 |  |
| Chenopodiaceae | *Atriplex barclayana* | 0.2 | 0 | **out** |
| Chenopodiaceae | *Arthrocnemum subterminale* | 0.2 | 0 | **out** |
| Chenopodiaceae | *Allenrolfea occidentalis* | 0.1 | 0 |  |
| Chenopodiaceae | *Suaeda cf. nigra* | 0.3 | 0.6 |  |
| Chenopodiaceae | *Atriplex canescens* | 0 | 0.4 | **in** |
| Chenopodiaceae | *Atriplex julacea* | 0.1 | 1 | **in** |
| Crassulaceae | *Dudleya cultrata* | 0.3 | 1 | **in** |
| Cucurbitaceae | *Marah macrocarpa* | 0 | 0.2 | **in** |
| Ephedraceae | *Ephedra californica* | 0 | 0.2 | **in** |
| Euphorbiaceae | *Euporbia misera* | 0 | 0.2 | **in** |
| Fabaceae | *Astragalus trichopodus* | 0 | 0.4 | **in** |
| Frankeniaceae | *Frankenia salina* | 0.7 | 0.2 | **out** |
| Sapindaceae | *Aesculus parryi* | 0 | 0.2 | **in** |
| Juncaceae | *Juncus acutus* | 0.6 | 0.2 | **out** |
| Malvaceae | *Sphaeralcea ambigua* | 0 | 0.4 | **in** |
| Nyctaginaceae | *Mirabilis laevis* | 0 | 0.4 | **in** |
| Plantaginaceae | *Gambelia juncea* | 0 | 0.6 | **in** |
| Poaceae | *Distichlis spicata* | 0.9 | 0.8 |  |
| Poaceae | *Distichlis littoralis* | 0.7 | 0.8 |  |
| Simmondsiaceae | *Simmondsia chinensis* | 0 | 0.2 | **in** |
| Solanaceae | *Lycium brevipes* | 0.8 | 0.6 |  |
| Solanaceae | *Lycium andersonii* | 0 | 0.2 | **in** |
| Solanaceae | *Lycium californicum* | 0.5 | 1 | **in** |

Table S3**:** Mean soil Electrical Conductivity (EC), and water-soluble cations (used to calculate sodicity) for midden and matrix sites at each region. EC is measured in μS/cm, all cations are measured in meq/L, sodicity is measured as a ratio.

|  |  | EC | Ca | K | Mg | Na | total | sodicity |  |
| --- | --- | --- | --- | --- | --- | --- | --- | --- | --- |
| Colonet | Matrix | 146.67 | 0.76 | 0.33 | 0.45 | 7.12 | 8.65 | 8.71 |  |
| Colonet | Midden | 143.33 | 1.10 | 0.40 | 0.42 | 1.69 | 3.61 | 3.04 |  |
| S-Quintin | Matrix | 35250.00 | 66.69 | 12.07 | 144.08 | 916.60 | 1139.45 | 109.23 |  |
| S-Quintin | Midden | 797.50 | 13.66 | 2.11 | 4.58 | 8.14 | 28.49 | 2.95 |  |
